# The Risk Factors and Predictors of Chronic Obstructive Pulmonary Disease in Patients With Obstructive Sleep Apnea–Hypopnea Syndrome at Plateau

**DOI:** 10.1111/crj.70053

**Published:** 2025-02-20

**Authors:** Yumei Geng, Yu Hu, Bin Li, Zhuoma Dawa, Fang Zhang

**Affiliations:** ^1^ Department of Respiratory and Critical Care Medicine Qinghai Provincial People's Hospital Xining China; ^2^ Department of Pharmacy Qinghai Provincial Traffic Hospital Xining China; ^3^ Qinghai Institute of Health Sciences Xining China; ^4^ Department of Science and Education Qinghai Provincial People's Hospital Xining China; ^5^ Suzhou Medical College of Soochow University Suzhou China; ^6^ Guide County People's Hospital Xining China

**Keywords:** COPD, OSAHS, plateau, risk factors

## Abstract

**Background:**

OSAHS patients with COPD (i.e., overlap syndrome) have a significantly worse prognosis than those with OSAHS alone, and the role of plateau hypoxia in the occurrence of the disease is still unclear. This underscores the urgent need to explore the risk factors for the incidence of COPD in patients with OSAHS and to identify predictors for the occurrence of overlap syndrome at plateau.

**Methods:**

Fifty patients with OSAHS and 34 patients with overlap syndrome were enrolled in this study. Demographic, auxiliary examination, and laboratory data were collected.

**Results:**

All patients enrolled were obstructive sleep apnea–hypopnea syndrome. The apnea–hypopnea index (AHI), the number of hypopnea, and the oxygen desaturation index were higher in the group of patients with overlap syndrome than in the group of patients with OSAHS. The mean pulse oxygen saturation was lower than that in the group of patients with OSAHS (*p* < 0.05). The right heart structure and function indexes (PASP, right atrial transverse diameter, RVTD, BNP, TNI) in patients with overlap syndrome were worse than those in patients with OSAHS (*p* < 0.05), and this worse cardiovascular status was positively correlated with inflammatory factors such as high‐sensitivity C‐reactive protein, IL‐6, and PCT (*p* < 0.05). Binary logistic regression analysis indicated that PASP, smoking index, and AHI were independent risk factors for OSAHS developing into overlap syndrome. ROC curve showed that the area under the curve of the combination of the three markers for predicting overlap syndrome was 0.908 (95% CI 0.843–0.974, *p* = 0.000), with a sensitivity of 0.882 and a specificity of 0.820. The optimal cutoff values for PASP were 42.5 mmHg, 15 for the smoking index, and 25.65 for the AHI based on the Youden index.

**Conclusions:**

Our study reveals that overlap syndrome has more frequent nighttime hypopnea and hypoxia than OSAHS alone. The cardiovascular complications of overlapping syndromes at plateau are more pronounced, possibly due to the exacerbation of the systemic inflammatory response. The combination of PASP, smoking index, and AHI can be a powerful tool for predicting and assessing the occurrence of COPD in OSAHS patients from plateau populations in China. These findings have the potential to significantly improve the management and prognosis of patients with overlap syndrome.

## Graphical abstract

Our study contributes to predict the incidence of COPD in patients with OSAHS at plateau, identify the independent risk factors, and determine three indicators for predicting OSAHS with COPD, which provide a scientific basis for clinicians to recognize COPD in OSAHS patients at an early stage.

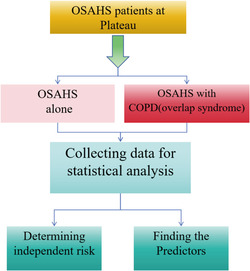

## 1 Introduction

Obstructive sleep apnea–hypopnea syndrome (OSAHS) is a chronic respiratory sleep disorder characterized by recurrent nocturnal upper airway obstruction and decreased ventilation in patients. The different durations and degrees of each obstruction cause different degrees of decreased blood oxygen saturation [1]. Chronic obstructive pulmonary disease (COPD) is a common preventable and treatable disease characterized by progressive airflow limitation. When COPD is in acute exacerbation, the increased airway resistance is more likely to lead to type II respiratory failure, which is life‐threatening [2].

Previous studies have shown that the incidence of COPD in OSAHS patients is about 1% in the general population [3], and the coexistence of COPD and OSAHS was first proposed as an overlap syndrome by David Flenley in 1985 [4]. Both have the same pathological changes in disease progression, which cause severe hypoxemia, a high risk of cardiovascular events, and, subsequently, poor prognosis [5]. Patients with overlapping syndromes have been found to have more severe sleep‐related hypoxemia and risk of hypertension, respiratory failure, cardiovascular accident, and cerebrovascular accident than those with COPD or OSAHS separately [6]. They have poorer quality of life [7], more frequent acute exacerbations of COPD [8], and higher morbidity and mortality rates [9]. As a result, patients with overlap syndrome have a more severe condition and worse prognosis, and early recognition is crucial to reduce the morbidity and mortality of overlap syndrome [10]. However, some of the patients with OSAHS lacked typical COPD symptoms, coupled with the fact that many healthcare professionals do not have an awareness of COPD assessment in patients with OSAHS, which led to some of the OSAHS being underdiagnosed [11, 12]. Therefore, the risk factors of OSAHS combined with COPD should be screened out, and early evaluation and intervention can improve the prognosis of patients with overlap syndrome.

The plateau region is a unique ecological environment system characterized by low atmospheric pressure, low partial pressure of oxygen, intense ultraviolet rays, and large temperature differences between day and night. Residents of the area with chronic respiratory diseases are prone to respiratory failure due to living in a low‐oxygen, low‐pressure environment for long periods [13, 14]. The hypoxia environment at plateau has a detrimental effect on human respiratory immunity and physiology, and it is well known that OSAHS and COPD are both chronic hypoxic lung diseases. However, it is still unclear whether plateau hypoxia can aggravate or protect the incidence of overlap syndrome, and further research is needed. Thus, we conducted a single‐center retrospective cross‐sectional study to clarify the clinical characteristics and risk factors of COPD in patients with OSAHS and to identify predictors for the occurrence of overlapping syndromes at plateau.

## 2 Methods

### 2.1 Study Design

Qinghai Provincial People's Hospital is one of the largest and best referral centers for the diagnosis and treatment of respiratory diseases on the Tibetan Plateau. All patients visiting our center for OSAHS between January 2019 and December 2022 were consecutively enrolled. Inclusion criteria were patients whose apnea–hypopnea index (AHI) ≥ 5 events per hour [15] and satisfied simultaneously the diagnostic criteria of the ratio of the forced the first second of expiratory volume to the forced vital capacity (FEV1/FVC) < 0.7 in the overlap syndrome group [16]. At the same time, the enrolled patient needs to fulfill the conditions for long‐term residence at plateau (2000 m above sea level). Exclusion criteria were patients who complicated with severe organ diseases such as liver, kidney, heart, or other life‐threatening diseases such as shock, cerebral hemorrhage, or severe complications such as pulmonary encephalopathy or gastrointestinal bleeding and who complicated with other lung diseases, such as asthma, severe pneumonia, pulmonary fibrosis, and lung cancer. Of these, 34 were in the group of patients with overlapping syndromes, and 50 were in the group of patients with OSAHS alone. Demographic and auxiliary examinations (polysomnography, pulmonary function test, color Doppler ultrasonography) and laboratory data were collected. The study protocol was approved by the medical ethics committee of Qinghai Provincial People's Hospital (number: 2022‐69).

### 2.2 Clinical Characteristics

Clinical characteristics of all participants, including gender, age, altitude of residence, BMI, and smoking index, were collected. BMI was calculated by dividing body weight in kilograms by height in square meters. The smoking index was equal to the number of cigarettes per day multiplied by the number of years of smoking.

### 2.3 Transthoracic Echocardiography

Echocardiography (Philips EPIQ 7C) was used to directly measure the right ventricular structural indices, including right atrial transverse diameter, the transverse diameter of the right ventricle (RVTD), right ventricular wall thickness (RVAW), and outflow tract of the right ventricle (RVOT). PASP was calculated by indirect measurement of peak velocity of tricuspid regurgitation (VTR) and inner diameter of the right pulmonary artery (RPA) by echocardiography. The calculation formula is as follows: PASP = 4V^2^TR + RPA.

### 2.4 Statistical Analysis

All data were analyzed by the SPSS 25.0 and GraphPad 6.0 software. The normal distribution data were presented as the mean standard deviation, and the skewed distribution data were expressed as the median (the first quartile and the third quartile). The two groups were compared by independent‐sample *t*‐test for normal distribution and equal variance data; no‐parametric Mann–Whitney test for skewed distribution data, sample rates, and composition ratios were compared using the chi‐square test. Spearman correlation was used to investigate the association between the different variables. Binary logistic regression analysis was used to evaluate the independent risk factors of overlap syndrome, and the receiver operating characteristic (ROC) curve was drawn to analyze the predictive value of independent risk factors for overlap syndrome. A *p*‐value of less than 0.05 was considered statistically significant.

## 3 Results

### 3.1 Characteristics of Study Participants

A total of 84 patients (50 patients with OSAHS alone and 34 patients with overlap syndrome) were included in this study. In the OSAHS group, there were 39 males and 11 females, aged 28–78 years, with an average age of 51.92 ± 10.16, while in the control group, there were 24 males and 10 females, aged 32–76 years, with an average age of 56.35 ± 10.44. There was no significant difference in sex or age between the two groups (*p* > 0.05). However, the altitude of residence and BMI in the overlap syndrome group were significantly lower than those in the OSAHS group, and the smoking index was higher than in the overlap syndrome group (*p* < 0.05), as laid out in Table 1.

**TABLE 1:** Demographic and clinical characteristics of participants.

**Variates**		**OSAHS (*n* = 50)**	**Overlap syndrome (*n* = 34)**	***t*/*Z*/** *χ* ^*2*^ **value**	***p***
Age (years)		51.92 ± 10.16	56.35 ± 10.44	−1.94	0.056
Sex (*χ* ^2^)				0.593	0.608
Male (*n* [%])		39 (78%)	24 (71%)		
Female (*n* [%])		11 (22%)	10 (29%)		
Altitude (m)		2609.50 (2261.00, 3045.00)	2261.00 (2261.00, 2494.50)	642.50	0.043
BMI (kg/m^2^)		29.00 (28.00, 31.30)	25.70 (23.65, 27.93)	1079.50	0.001
Smoking index		0.00 (0.00125.00)	375.00 (100.00, 525.00)	1254.00	0.000
Comorbidity (*χ* ^2^)	Hypertension (*n* [%])	11 (48%)	11 (46%)	1.122	0.320
	Diabetes (*n* [%])	12 (24%)	11 (46%)	0.710	0.459
	Others: liver cysts, kidney cysts or other diseases that do not affect heart and lung function(*n* [%])	9 (18%)	8 (24%)	0.383	0.587
**Auxiliary examination**					
Polysomnography data	AHI	17.35 (10.35, 31.33)	30.70 (12.80, 55.80)	1147.00	0.007
	Obstructive apneas (*n*)	9.50 (1.75, 32.75)	9.00 (2.00, 136.25)	990.50	0.199
	Obstructive hypopneas (*n*)	80.00 (48.50123.25)	105.50 (70.75, 200.25)	1095.00	0.026
	Central sleep apneas (*n*)	0.00 (0.00, 2.25)	0.50 (0.00, 3.25)	935.00	0.396
	Mixed sleep apneas (*n*)	0.00 (0.00, 0.00)	0.00 (0.00, 1.25)	968.50	0.132
	Oxygen desaturation index	27.10 (17.28, 39.73)	50.65 (29.28, 63.03)	1149.50	0.006
	Mean pulse oxygen saturation	0.86 (0.80, 0.89)	0.82 (0.76, 0.86)	553.50	0.007
	Arousal index (times/h)	30.75 (17.80, 46.23)	26.95 (15.68, 38.23)	700.00	0.172
FEV1 (L)		2.63 ± 0.77	1.24 ± 0.36	9.80	0.000
FEV1/FVC		0.81 (0.75, 0.85)	0.54 (0.38, 0.67)	657.00	0.000
PASP (mmHg)		35.50 (29.75, 35.50)	50.00 (40.00, 74.50)	1414.50	0.000
Right atrial transverse diameter (mm)		34.50 (32.00, 34.50)	41.00 (35.00, 47.25)	1161.00	0.001
RVTD (transverse diameter of the right ventricle [mm])		29.00 (26.00, 31.00)	33.00 (28.75, 37.25)	1155.00	0.001
RVAW (right ventricular wall thickness [mm])		4.00 (4.00, 5.00)	4.00 (4.00, 5.00)	875.00	0.528
RVOT (outflow tract of right ventricle [mm])		30.00 (28.00, 33.00)	30.00 (27.00, 36.00)	842.50	0.802
LVEF (left ventricular ejection fraction [%])		64.63 ± 6.42	64.00 ± 5.71	0.45	0.651
**Laboratory examination**					
Red blood cell (*10^12^/L)		5.59 (5.00, 6.23)	5.79 (4.95, 6.62)	870.500	0.852
Number of red cell distribution width variations (%)		14.30 (12.80, 14.30)	15.55 (13.65, 17.65)	984.00	0.222
Mean corpuscular hemoglobin (pg)		30.35 (29.18, 31.45)	31.05 (29.40, 32.33)	999.50	0.173
Mean corpuscular hemoglobin concentration (g/L)		330.00 (324.00, 340.25)	330.50 (315.75, 337.25)	699.00	0.169
Hemoglobin (g/L)		167.00 (153.00, 191.25)	172.00 (154.00, 206.25)	941.00	0.407
Hematocrit (%)		50.40 (45.85, 57.53)	53.40 (47.63, 62.75)	955.00	0.339
Creatine kinase (U/L)		55.00 (39.50, 95.50)	59.00 (30.75, 95.00)	806.00	0.802
Creatine kinase‐MB (CK‐MB [U/L])		10.00 (8.00, 13.00)	11.50 (9.00, 15.00)	1040.00	0.054
Lactate dehydrogenase (LDH [U/L])		208.00 (176.00, 254.50)	219.50 (183.75, 287.50)	966.50	0.288
Lactate dehydrogenase 1 (LDH1 (U/L])		36.00 (29.00, 50.25)	43.00 (35.00, 62.50)	1023.00	0.065
α‐Hydroxybutyrate dehydrogenase (U/L)		129.00 (104.75, 156.75)	142.00 (111.00, 182.50)	1004.50	0.095
Troponin I (TNI [Pg/mL])		1.74 (0.32, 5.22)	5.53 (1.68, 10.65)	1109.50	0.008
Serum iron (μmol/L)		16.31 ± 9.91	16.37 ± 9.25	−0.028	0.978
Unsaturated iron binding capacity (μmol/L)		42.16 ± 15.98	36.39 ± 15.64	1.64	0.105
Total iron binding capacity (μmol/L)		52.27 ± 13.38	51.05 ± 11.78	0.43	0.670
Transferrin saturation		0.25 (0.17, 0.39)	0.31 (0.15, 0.43)	909.50	0.588
Ferritin (ng/mL)		178.20 (94.49, 506.9)	133.35 (60.71, 291.48)	438.50	0.245
Creatinine (μmol/L)		74.00 (67.00, 86.00)	71.50 (62.50, 85.25)	811.00	0.722
High‐sensitivity C‐reactive protein (mg/dL)		0.25 (0.13, 0.79)	0.43 (0.22, 1.12)	1006.00	0.155
Brain natriuretic peptide[BNP (pg/mL)]		38.00 (19.00, 99.75)	162.00 (41.50, 509.00)	1232.00	0.000
Erythrocyte sedimentation rate (mm/h)		2.00 (1.50, 5.00)	2.00 (1.00, 3.25)	771.00	0.552
pH value		7.42 ± 0.01	7.42 ± 0.01	−0.118	0.907
PaCO_2_ (mmHg)		39.00 (35.00, 43.25)	39.00 (36.00, 43.00)	858.50	0.938
PaO_2_ (mmHg)		58.00 (52.00, 66.00)	61.50 (47.50, 77.50)	892.00	0.702
Oxygenation index (PaO_2_/FiO_2_)		276.19 (247.62314.29)	134.48 (124.14, 148.28)	601.00	0.000
SaO_2_ (%)		90.00 (87.00, 93.00)	91.50 (83.00, 94.00)	868.00	0.869
Serum potassium concentration		3.89 ± 0.35	3.83 ± 0.479	−0.688	0.493
Serum sodium concentration		138.96 ± 3.52	139.00 ± 2.40	0.058	0.954

### 3.2 Polysomnographic Characteristics of Study Participants

**FIGURE 1:**
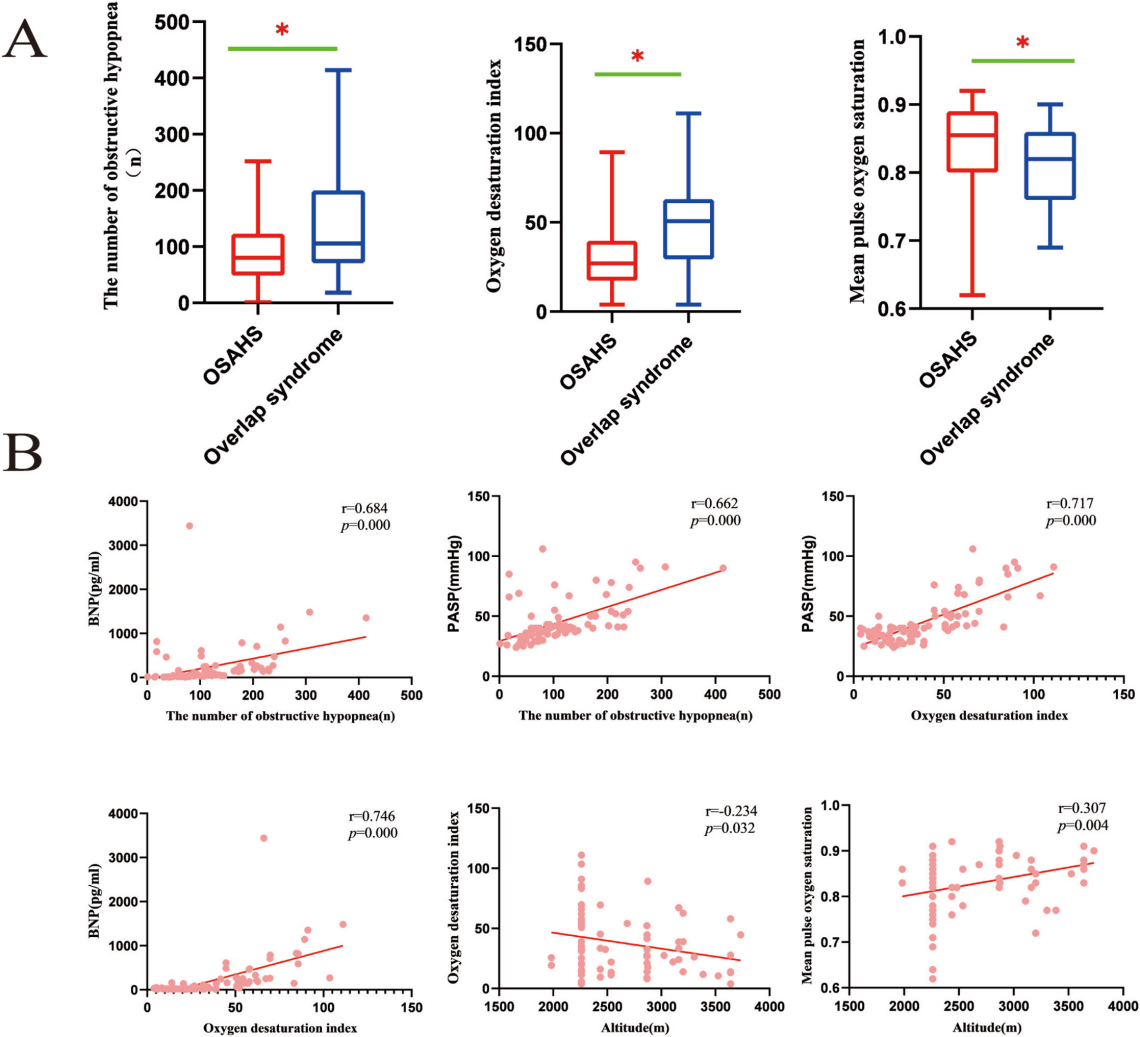
(A) Polysomnographic characteristics in subjects with OSAHS and those with overlap syndrome. (B) Spearman correlations between altitude, polysomnographic parameters, and clinical variables in patients with OSAHS and overlap syndrome.

The type of sleep apnea was predominantly obstructive in both groups of patients enrolled, with the AHI of patients in the overlap syndrome group being higher than that of the group of patients with OSAHS, and the sleep monitoring performance of both groups having been predominantly obstructive hypopnea followed by obstructive apnea, with central and mixed apnea being less common. The number of obstructive hypopnea and oxygen desaturation index were higher in the overlap syndrome group than in the OSAHS group. In contrast, the mean pulse oxygen saturation was lower than in the OSAHS group(*p* < 0.05), as laid out in Table 1 and Figure 1A. As shown in Figure 1B, the number of obstructive hypopnea was positively correlated with BNP(*r* = 0.684, *p* = 0.000) and PASP(*r* = 0.662, *p* = 0.000) and the oxygen desaturation index was positively correlated with BNP(*r* = 0.746, *p* = 0.000) and PASP (*r* = 0.717, *p* = 0.000). Altitude was negatively correlated with the oxygen desaturation index(*r* = −0.234, *p* = 0.032) and positively correlated with the mean pulse oxygen saturation (*r* = 0.307, *p* = 0.004).

### 3.3 Comparison of Clinical Parameters Between the two Groups

**FIGURE 2:**
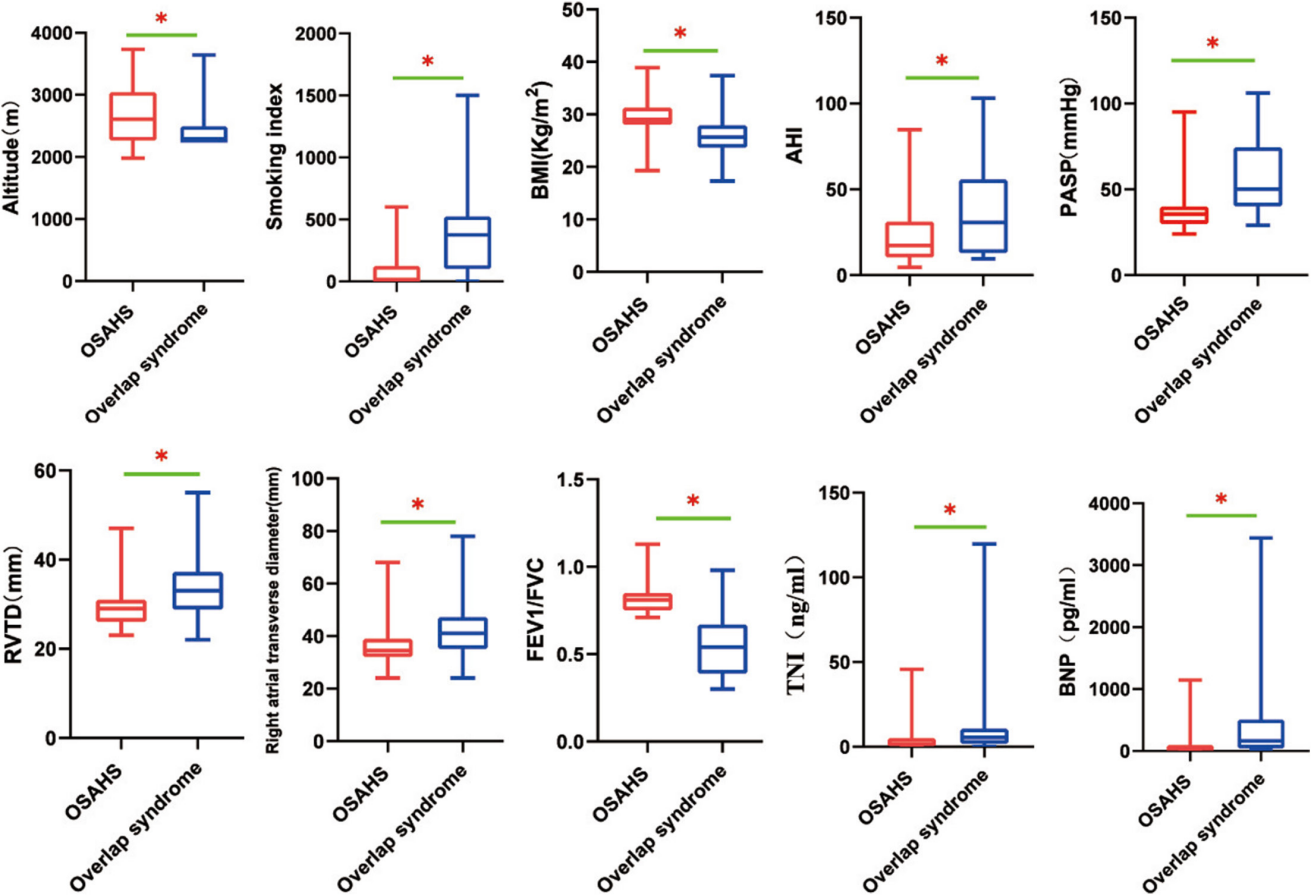
Comparison of clinical parameters between subjects with OSAHS and those with overlap syndrome (red represents the OSAHS group, and blue represents the overlap syndrome group).

Patients with overlap syndrome had lower FEV1/FVC than those with OSAHS alone, which was consistent with the inclusion criteria. However, the AHI and PASP in the overlap syndrome group were higher than those in the OSAHS group; the right ventricular structure and function parameters (such as RVTD, right atrial transverse diameter, TNI, BNP) in the overlap syndrome group were worse than that in the OSAHS group(*p* < 0.05), as shown in Figure 2.

### 3.4 Spearman Correlations Between Altitude, FEV1/FVC Levels and Clinical Variables in Patients With OSAHS and Overlap Syndrome

**FIGURE 3:**
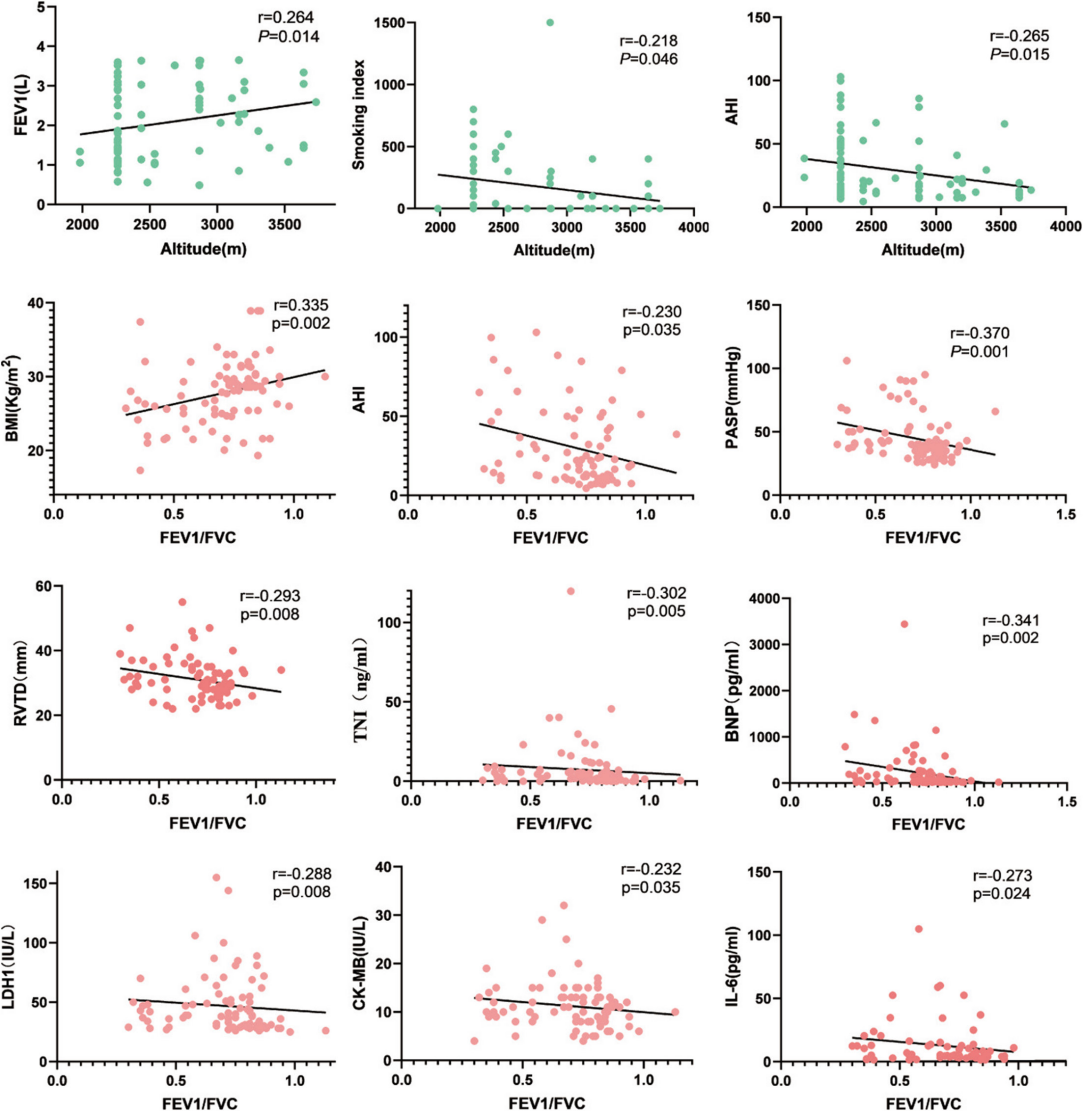
Spearman correlations between altitude, FEV1/FVC levels and clinical variables in patients with OSAHS and overlap syndrome.

As shown in Figure 3, the altitude was positively correlated with FEV1(*r* = 0.264, *p* = 0.014) and negatively correlated with smoking index(*r* = −0.218, *p* = 0.046) and AHI(*r* = −0.265, *p* = 0.015). FEV1/FVC was positively correlated with BMI (*r* = 0.335, *p* = 0.002) and negatively correlated with AHI (*r* = −0.230, *p* = 0.035), PASP (*r* = −0.370, *p* = 0.001), RVTD (*r* = −0.293, *p* = 0.002), TNI (*r* = −0.302, *p* = 0.005), BNP (*r* = −0.341, *p* = 0.002), LDH1 (*r* = −0.288, *p* = 0.008), CK‐MB (*r* = −0.232, *p* = 0.035), and IL‐6 (*r* = −0.273, *p* = 0.024).

### 3.5 Spearman Correlations Between PASP and Clinical Variables in Patients With OSAHS and Overlap Syndrome

**FIGURE 4:**
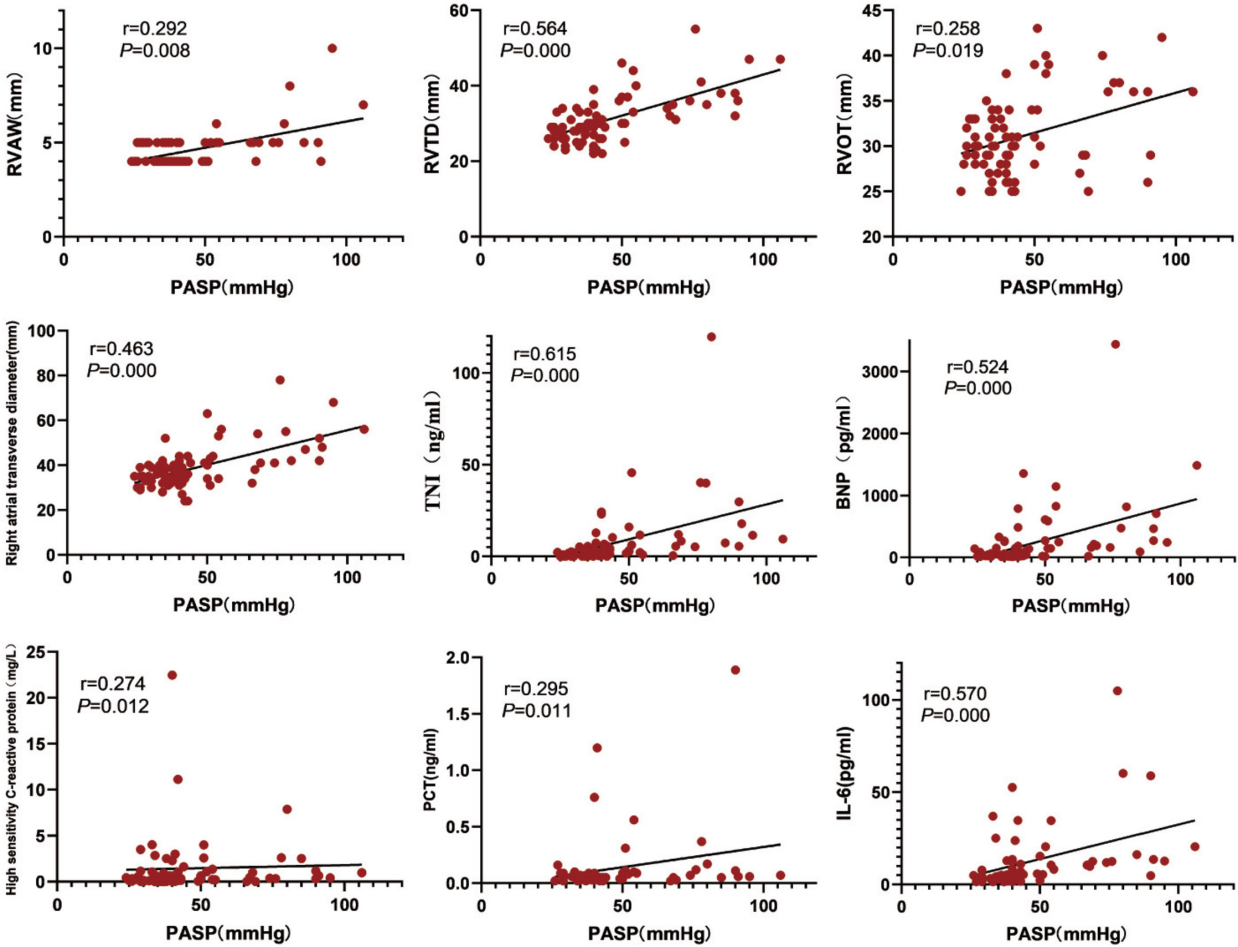
Spearman correlations between PASP and clinical variables in patients with OSAHS and overlap syndrome.

As shown in Figure 4, PASP was positively correlated with the right ventricular structure and function parameters (RVAW *r* = 0.292, *p* = 0.008; RVTD *r* = 0.564, *p* = 0.000; RVOT *r* = 0.258, *p* = 0.019; right atrial transverse diameter *r* = 0.463, *p* = 0.000; TNI *r* = 0.615, *p* = 0.000; BNP *r* = 0.524, *p* = 0.000), and inflammatory factors (high‐sensitivity C‐reactive protein *r* = 0.274, *p* = 0.012; IL‐6 *r* = 0.570, *p* = 0.000; PCT *r* = 0.295, *p* = 0.011).

### 3.6 Binary Logistic Regression Analyses to Identify the Independent Risk Factors for COPD in OSAHS Patients

Binary logistic regression analysis was performed according to whether OSAHS was combined with COPD (no = 0, yes = 1) as the dependent variable and altitude, BMI, smoking index, AHI, PASP, right atrial transverse diameter, RVTD, TNI, and BNP as the independent variables. The results suggest that PASP, smoking index, and AHI are independent risk factors for overlap syndrome (Table 2).

**TABLE 2:** Odds ratios for the presence of overlap syndrome derived from binary logistic regression analyses.

Variates	Regression coefficient	Standard error	Wald *X* ^2^ value	*p*	OR value	95% CI
PASP	0.076	0.022	11.921	0.001	1.079	1.033–1.126
Smoking index	0.006	0.002	12.564	0.000	1.006	1.003–1.009
AHI	0.036	0.015	6.084	0.014	1.037	1.007–1.067

### 3.7 ROC Curve Analyses of the Independent Risk Factors for Predicting Overlap Syndrome

**FIGURE 5:**
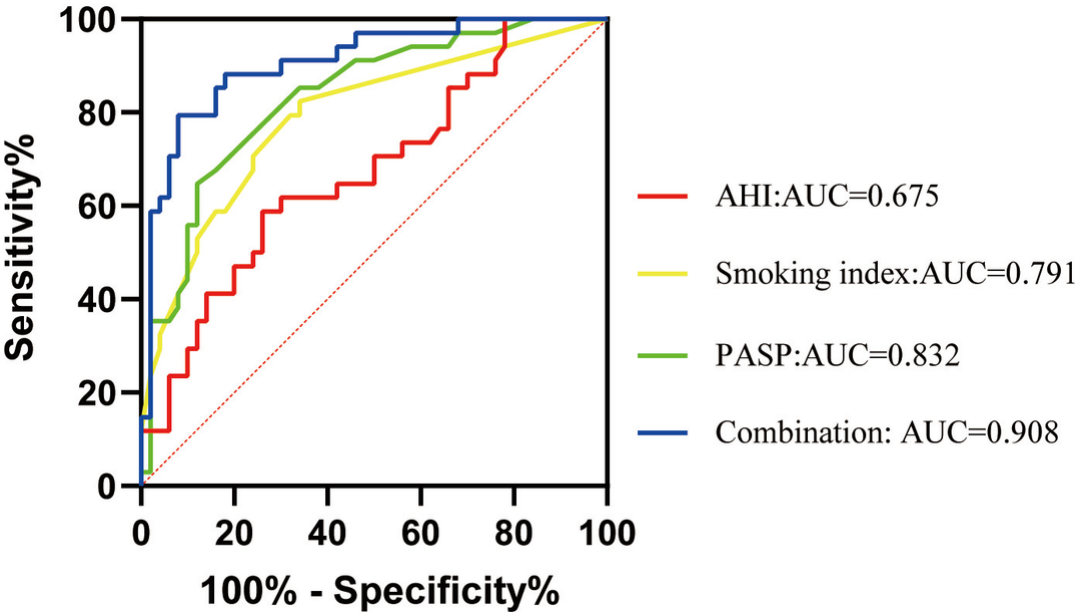
Receive operating characteristic analysis of the independent risk factors in predicting overlap syndrome.

ROC curve analyses of the independent risk factors for predicting overlap syndrome are shown in Figure 5. PASP, smoking index and AHI had specific predictive values for overlap syndrome. The curve area was 0.832 (95% CI 0.744–0.920, *p* = 0.000), 0.791 (95% CI 0.691–0.892, *p* = 0.000) and 0.651 (95% CI 0.558–0.792, *p* = 0.007), respectively. The optimal cutoff value of PASP calculated by Youden index was 42.5 mmHg, with a sensitivity of 64.7% and a specificity of 88.0%. The optimal cutoff value of the smoking index was 15, with a sensitivity of 82.4% and a specificity of 66.0%. The best cutoff value of AHI was 25.65, with a sensitivity of 58.8% and a specificity of 74.0%. The area under the curve of the combination of the three markers for predicting overlap syndrome was 90.8% (95% CI 0.843–0.974, *p* = 0.000), the sensitivity was 88.2%, and the specificity was 82.0%. The cutoff values for PASP, smoking index, and AHI were 40 mmHg, 350, and 9.5 events/h, respectively. The relevant parameters of ROC curve analyses are summarized in Table 3.

**TABLE 3:** Receiver operating characteristic curve analyses of variates for overlap syndrome.

Variates	AUC (95% CI)	*p*	Optimal cutoff	Sensitivity	Specificity	Youden index
AHI	0.675 (0.558–0.792)	0.0068	25.65	0.588	0.740	0.328
Smoking index	0.791 (0.691–0.892)	< 0.0001	15	0.824	0.660	0.484
PASP	0.832 (0.744–0.920)	< 0.0001	42.5	0.647	0.880	0.527
The combination of the three indexes	0.908 (0.843–0.974)	< 0.0001	PASP (40 mmHg), smoking index (350), AHI (9.5);	0.882	0.820	0.702

## 4 Discussion

The prevalence of overlap syndrome, that is, the coexistence of two diseases, COPD and OSAHS, ranges from 10% to 65% according to epidemiological surveys due to differences in epidemiological survey methodology and geographic sampling [8, 17, 18, 19]. However, little research has been carried out on the pathophysiology and clinical outcomes of overlap syndrome. Previous studies have shown that COPD and OSAHS share similar physiological and molecular mechanisms, such as hypoxia and systemic inflammation, both of which can lead to cardiovascular and other systemic complications. Patients with overlap syndrome experience more severe oxygen desaturation during sleep and a higher incidence of respiratory insufficiency than patients with OSAHS alone [20]. However, this is exacerbated by the complex interplay between hypoxemia and inflammatory mediators in the overlap syndrome, which has been considered to explain the higher mortality rate than COPD or OSAHS alone in recent years [21]. Therefore, our study aimed to identify the risk factors for COPD in OSAHS and to determine the predictors for clinicians' early recognition of overlap syndrome, which is essential for improving the prognosis of overlap syndrome. Our retrospective analysis revealed that patients with plateau OSAHS had predominantly obstructive sleep apnea, and polysomnography showed a significant increase in the number of hypopnea and oxygen desaturation index and a decrease in mean pulse oxygen saturation, which was more pronounced in the overlap syndrome. The number of hypopnea and oxygen desaturation index were positively correlated with BNP and PASP. The group of patients with overlap syndrome had worse right heart function parameters than the group with OSAHS alone, and this may be related to higher inflammatory factors (hypersensitive C‐reactive protein, PCT, IL‐6). ROC curve analysis suggested that AHI, smoking index, and PASP were independent risk factors for the development of COPD in patients with OSAHS.

The incidence of respiratory diseases is increased at plateau due to the unique geographical environment of low pressure, hypoxia, cold, and dryness [22]. However, there is no epidemiological data related to the overlap syndrome at plateau. In this study, we found that patients with overlap syndrome lived at a lower average altitude than patients with OSAHS alone. Previous studies consistent with our findings have shown that altitude is not only unrelated but even inversely related to the incidence and severity of chronic lung diseases such as COPD [23, 24]. This may be the result of respiratory failure, sleep apnea, impaired respiratory function, and exacerbation of respiratory disease in lowlanders who presented hypoxic maladaptation due to rushing into the plateau [25, 26]. However, long‐term highlanders have adapted and tolerated low‐pressure hypoxia, and the response to hypoxia ventilation is blunted [27], resulting in the higher altitude, the higher FEV1, the lower AHI found in our study, coupled with the lower smoking index. Therefore, plateau hypoxia may play a protective role in the occurrence of COPD in OSAHS, yet further support from the large sample of epidemiological data is needed in the future.

Altitude can have dramatic short‐ and long‐term effects on respiratory regulation during sleep. The effects of high altitude on sleep are primarily characterized by hypobaric hypoxia triggering respiratory compensation and gradual adaptation, which usually occurs over days to weeks. These effects alter breathing patterns and increase hourly apnea and hypopnea indices, leading to periodic breathing (PB) [28, 29]. Nocturnal PB can cause frequent awakenings and lead to fragmentation of sleep structure [30]. With prolonged time at high altitude, the adaptability of the body to long‐term hypoxia gradually improves, but poor subjective sleep quality, sleep alterations dominated by frequent awakenings, and pronounced nocturnal hypoxemia are still commonly experienced by high‐altitude residents [31]. Our study showed that patients with OSAHS at plateau had poor sleep quality, a high arousal index, and sleep dominated by obstructive hypopnea alternating with obstructive apnea accompanied by significant hypoxemia, in line with the findings of the previous study. However, patients with overlap syndrome have high airway resistance and airflow limitation due to the presence of COPD [32], a small airway obstructive lung disease, resulting in an increased number of obstructive hypopnea and lower mean pulse oxygen saturation. Interestingly, with the increase in altitude, the oxygen desaturation index decreased, and the mean pulse oxygen saturation increased. This indirectly confirms the previous results that patients with overlap syndrome are more severely ill but at lower altitude than patients with OSAHS. This may be attributed to the fact that people living at higher altitudes are mostly Tibetans, who have specific genes for adaptation to hypoxia, such as EPAS1, EGLN1, and PPARA [33], and who have tolerated and adapted to hypoxia and are blunted to hypoxia, thus showing oxygen desaturation index and mean pulse oxygen saturation that does not correspond to the performance of altitude.

Previous studies have suggested that smoking is one of the critical risk factors for COPD [34], and the results of our study are consistent with the conclusions of previous studies. The smoking index of overlap syndrome patients is significantly higher than that of OSAHS patients alone, and the smoking index is one of the independent risk factors for overlap syndrome, which suggests that clinicians should provide smoking cessation interventions for OSAHS patients at an early stage in order to avoid the progression of the disease. The abnormal structure of the pharyngeal cavity caused by the obese body type is one of the crucial factors in the pathogenesis of OSAHS. The results of this study showed that the BMI of patients with overlap syndrome was lower than that of OSAHS patients alone, which may be related to chronic nutritional consumption of COPD patients over a long period [35].

Cardiovascular complications are prevalent complications of respiratory diseases, and pulmonary hypertension and cor pulmonale are common complications of OSAHS and overlap syndrome [36, 37]. Our study showed that patients with overlap syndrome had significantly higher PASP and worse right ventricular structure and function parameters (right atrial transverse diameter, RVTD, BNP, TNI) than patients with OSAHS alone, which indicated the poor outcome of right heart function in patients with overlap syndrome. We also found that PASP is one of the independent risk factors for overlap syndrome. This shows that pulmonary hypertension is both a cause and a consequence of overlap syndrome, and it may form a circular loop of interaction with disease progression. At the same time, we found that PASP was positively correlated with inflammatory factors (high‐sensitivity C‐reactive protein, PCT, IL‐6). The higher the PASP was, the more severe the systemic inflammatory response was, which contributes more to the development of overlap syndrome, leading to worse cardiovascular status [38, 39]. It is well known that COPD is associated with an abnormal inflammatory response of the lung to harmful gases or harmful particles. Consistent with this conclusion, our study showed that both FEV1/FVC and PASP were positively correlated with inflammatory factors to a certain extent, which suggests that an essential mechanism for the occurrence of COPD in OSAHS may be the involvement of inflammatory factors and provides new ideas for future molecular experimental studies.

The status of lung function determines the incidence of cardiovascular complications [40]. Our study showed that FEV1/FVC was negatively correlated with PASP, right ventricular structure, and function parameters; this suggests that declining lung function increases cardiovascular risk. At the same time, FEV1/FVC is negatively correlated with AHI, suggesting that the worse the lung function is, the more severe the intermittent hypoxia of the individual is, which further aggravates the system's hypoxic state. However, it was positively correlated with BMI, further suggesting that nutritional depletion is more pronounced in patients with poor lung function [41].

To the best of our knowledge, this is the first clinical study conducted to investigate the predictors of combined COPD in OSAHS in highland areas. We found that the ROC area of PASP, AHI, and smoking index combined to predict overlap syndrome in OSAHS patients was larger, and the predictive value was higher, whereas more extensive cohort studies are needed in the future to evaluate further the value of the combined prediction of the three indicators. Undeniably, there are some limitations in this study. Firstly, the sample size of the study was not large enough, resulting in the correlation coefficients in the correlation analysis not being very high. Secondly, this study was a retrospective analysis, making it impossible to collect subjective data such as patients' self‐reported sleep and daytime sleepiness levels. However, the results of this study are fascinating and provide new ideas for future treatment of overlap syndrome.

In conclusion, our study collected cases of OSAHS and overlap syndrome analyzed sleep characteristics of OSAHS patients at plateau, screened out the clinical factors with significant differences between the two groups by statistical analysis, and further revealed that PASP, AHI, and smoking index were independent risk factors for overlap syndrome in highland areas which provides favorable evidence for exploring the pathogenesis of overlap syndrome patients and searching for new intervention targets. It also provides timing suggestions for clinicians to make early diagnoses and interventions.

## Author Contribution

Yumei Geng had the idea and designed the study. Fang Zhang supervised the study. Yu Hu and Bin Li did the statistical analysis. Zhuoma Dawa verified statistical analysis methodology and results. All authors contributed to the acquisition, analysis, or interpretation of data. Yumei Geng wrote the draft report. All authors have agreed to submit the manuscript with no conflicts of interest regarding its content.

## Ethics Statement

The study protocol was approved by the medical ethics committee of Qinghai Provincial People's Hospital (number: 2022‐69).

## Consent

Because this retrospective study was based on data from patient medical records, written informed consent was not obtained from the participants.

## Conflicts of Interest

The authors declare no conflicts of interest.

## Data Availability

Data sharing is not applicable to this article as no datasets were generated or analyzed during the current study.
